# Endoscopic treatment of bile duct stones after bariatric Roux-en-Y gastric bypass through endoscopic ultrasound-directed transgastric ERCP

**DOI:** 10.1055/a-2161-3450

**Published:** 2023-09-21

**Authors:** Francisco Vara-Luiz, Gonçalo Nunes, Pedro Pinto-Marques, Carla Oliveira, Ivo Mendes, Marta Patita, Jorge Fonseca

**Affiliations:** 1 Gastroenterology Department, Hospital Garcia de Orta, Almada, Portugal; 2 Egas Moniz Center for Interdisciplinary Research (CiiEM), Egas Moniz School of Health and Science, Caparica, Portugal

Endoscopic retrograde cholangiopancreatography (ERCP) is technically challenging after Roux-en-Y gastric bypass (RYGB) 1 . Standard approaches include enteroscopy-assisted and laparoscopy-assisted ERCP, which present difficult implementation in clinical practice 2 . The authors report endoscopic ultrasound-directed transgastric ERCP (EDGE) for the treatment of bile duct stones in RYGB patients ( Video 1 ).

**Video 1** Endoscopic ultrasound-directed transgastric ERCP (EDGE) used to successfully treat bile duct stones after Roux-en-Y gastric bypass.


A 62-year-old man was admitted with fever and abdominal pain. Past medical history was relevant for RYGB and small bowel resection due to mesenteric ischemia. Abdominal computed tomography (CT) was consistent with choledocholithiasis and acute cholecystitis. For biliary drainage the patient underwent ERCP using a pediatric colonoscope, but selective biliary cannulation was not achieved with a forward-viewing instrument. Considering the altered anatomy, EDGE was proposed.

Using a linear echoendoscope in the gastric pouch, EUS-guided puncture of the excluded stomach was accomplished with a 19G needle. Saline, methylene blue, and iodate contrast were injected allowing gastric fold visualization and lumen distension. A 20-mm lumen-apposing metal stent (LAMS) (Hot AXIOS; Boston Scientific, Marlborough, Massachusetts, USA) was successfully deployed creating a gastro-gastrostomy ( Fig. 1 ).

**Fig. 1:**
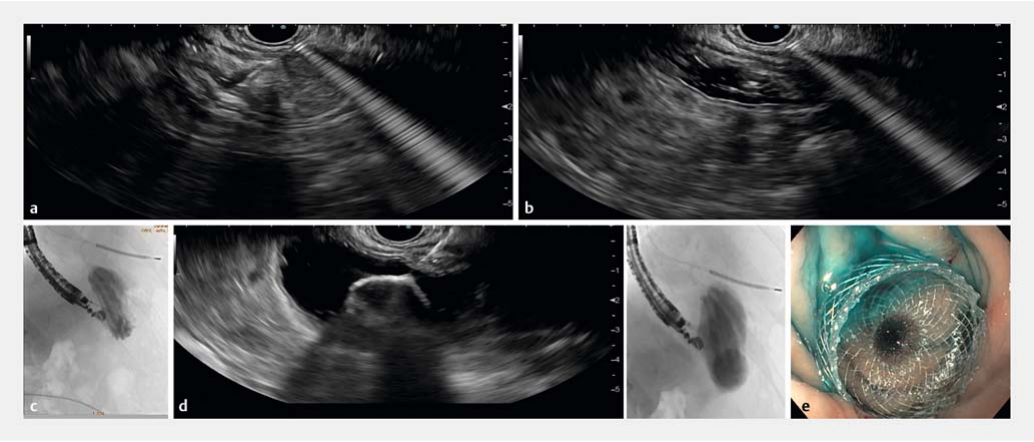
Endoscopic ultrasound (EUS)-guided placement of lumen-apposing metal stent (LAMS). **a** Puncture of the excluded stomach with a 19G needle. **b** Saline, methylene blue, and iodate contrast instillation allowing lumen distension. **c** Fluoroscopy of gastric folds. **d** Deployment of 20-mm LAMS under EUS and fluoroscopic view. **e** Methylene blue confirming successful gastro-gastrostomy.

After 7 days, anterograde progression to the papilla with a duodenoscope ( Fig. 2 ) was possible. Biliary cannulation was successful using the double guidewire technique, and endoscopic sphincterotomy was safely performed. Several biliary stones were removed with a Dormia basket and extraction balloon (15 mm). A 5-Fr pancreatic stent was placed to prevent post-ERCP pancreatitis. No procedural complications were observed and the LAMS was endoscopically removed after 4 weeks and the gastric fistula closed using argon plasma coagulation and a 9-mm over-the-scope clip ( Fig. 3 ). The patient remained asymptomatic after cholecystectomy.

**Fig. 2:**
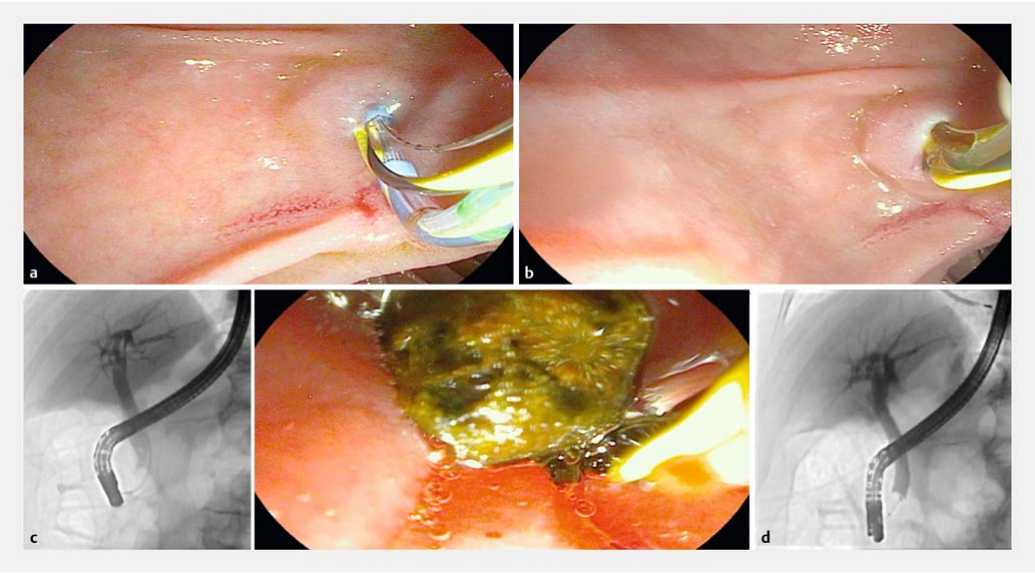
Transgastric endoscopic retrograde cholangiopancreatography. **a** Biliary cannulation using the double guidewire technique. **b** Endoscopic sphincterotomy. **c** Biliary stones removed under fluoroscopic and endoscopic view. **d** Final cholangiogram without common bile duct stones.

**Fig. 3:**
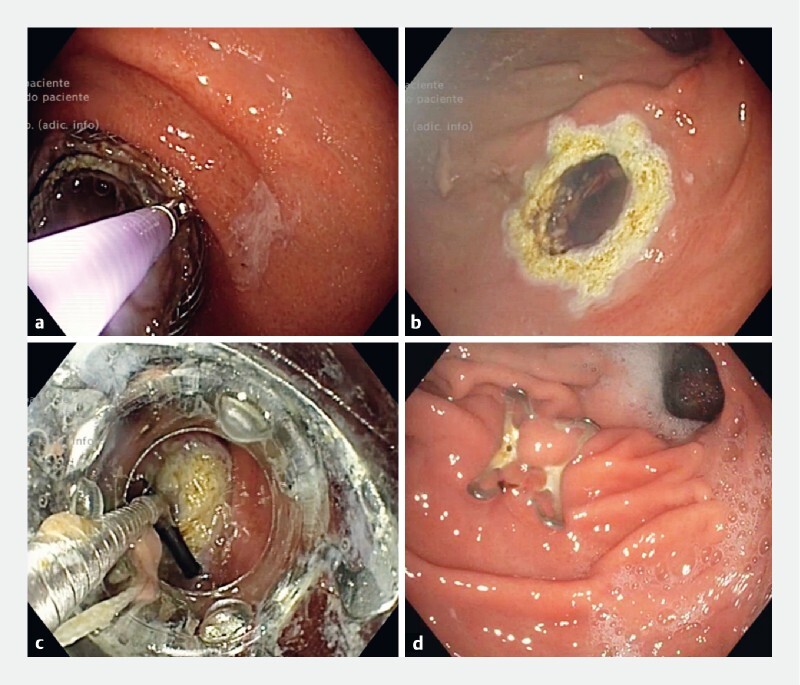
Gastro-gastrostomy closure. **a** LAMS removal with grasping forceps. **b** Argon plasma coagulation applied to the tract. **c** Gastric fistula closure using an over-the-scope clip (OTS-clip). **d** OTS-clip in situ.

The advantages of EDGE include its higher success rate and lower invasiveness, shortening hospitalization compared with endoscopy- and laparoscopy-assisted ERCP 3 4 . It is suggested as a first-line approach in expert centers. The authors exemplify the effectiveness and safety of this technique to treat pancreaticobiliary disorders after RYGB.

Endoscopy_UCTN_Code_TTT_1AS_2AD

## Notes

Endoscopy E-Videos

https://eref.thieme.de/e-videos
*E-Videos* is an open access online section of the journal *Endoscopy* , reporting on interesting cases and new techniques in gastroenterological endoscopy. All papers include a high-quality video and are published with a Creative Commons CC-BY license. Endoscopy E-Videos qualify for HINARI discounts and waivers and eligibility is automatically checked during the submission process. We grant 100% waivers to articles whose corresponding authors are based in Group A countries and 50% waivers to those who are based in Group B countries as classified by Research4Life (see: https://www.research4life.org/access/eligibility/ ). This section has its own submission website at https://mc.manuscriptcentral.com/e-videos
